# CD24-Siglec interactions in inflammatory diseases

**DOI:** 10.3389/fimmu.2023.1174789

**Published:** 2023-05-09

**Authors:** Yang Liu, Pan Zheng

**Affiliations:** OncoC4, Inc., Rockville, MD, United States

**Keywords:** CD24, COVID-19, graft vs host diseases, immunotherapy-related adverse events, Siglecs

## Abstract

CD24 is a small glycosylphosphatidylinositol (GPI)-anchored glycoprotein with broad expression in multiple cell types. Due to differential glycosylation, cell surface CD24 have been shown to interact with various receptors to mediate multiple physiological functions. Nearly 15 years ago, CD24 was shown to interact with Siglec G/10 to selectively inhibit inflammatory response to tissue injuries. Subsequent studies demonstrate that sialylated CD24 (SialoCD24) is a major endogenous ligand for CD33-family of Siglecs to protect the host against inflammatory and autoimmune diseases, metabolic disorders and most notably respiratory distress in COVID-19. The discoveries on CD24-Siglec interactions propelled active translational research to treat graft-vs-host diseases, cancer, COVID-19 and metabolic disorders. This mini-review provides a succinct summary on biological significance of CD24-Siglec pathway in regulation of inflammatory diseases with emphasis on clinical translation.

## Introduction

As a GPI-anchored glycoprotein, CD24 is expressed on multiple cell types in normal tissues, including hematopoietic and immature neuronal cells and tissue stem cells (1–3). While CD24 was first identified as a valuable marker for cellular development and differentiation, accumulating studies have revealed critical roles for CD24 in various pathological conditions, including autoimmune diseases (4–8), sepsis (9), metabolic disorders (10–12), graft vs host diseases (13, 14) and cancer (1, 15–20). Given the heterogeneity of in post-translational modifications of CD24, it is not surprising that CD24 may mediate different functions depending on its interacting partners. For example, fucosylated CD24 has been shown to be a ligand for P-selectin (21, 22), while sialylated CD24 have been shown to interact with Siglecs (9, 23). In this minireview, we will focus on CD24-Siglec interactions, with emphasis on potential translation of this pathway for patient care.

## CD24-Siglec interaction negatively regulates host responses to tissue injuries

Sialic acid-binding immunoglobulin-type lectins (Siglecs) constitute a subfamily of type I lectin. The first member of Siglecs, or Siglec 1 was reported nearly 40 years ago (24, 25). Fifteen human Siglecs have been subsequently characterized (26, 27). Among them, a subgroup group called CD33 family have attracted most attention as potential negative regulators of immune response as they have one or more ITIM-like domains that are phosphorylated upon ligand engagement, leading recruitment of Src homology 2 domain containing protein tyrosine phosphatase (SHP)1/2 and potentially suppress NFkB activation (26, 27). While Siglecs have selectivity for different sialoglycans (28), the endogenous ligands for Siglecs have not been identified decades after first description of Siglecs. Lack of endogenous ligand made it difficult to discern the physiological function of Siglecs.

In 2009, Chen et al. reported CD24 as the major endogenous ligand for Siglec 10 and its mouse homologue Siglec G (23). The interaction was demonstrated by direct binding *in vitro* and co-precipitation in immune cells. Subsequent studies demonstrate requirement for sialylation in the interaction (9).

**Figure 1:**
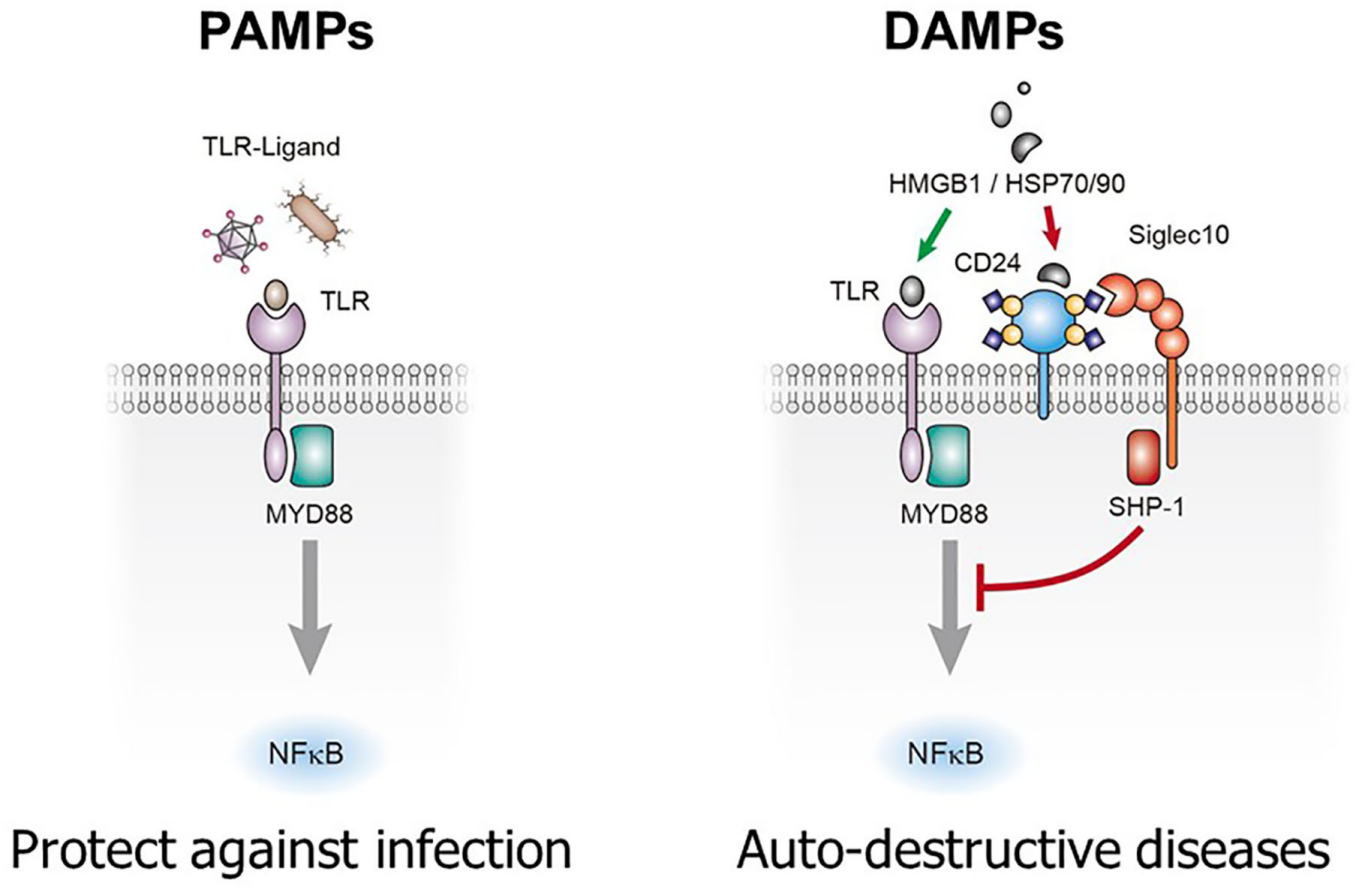
CD24-Siglec 10/G interaction selectively repress inflammatory response to tissue injuries. Sialylated CD24 interacts with Siglec 10 to repress inflammatory responses to danger (damage)-associated molecular patters (DAMPs) but not those to pathogen-associated molecular patterns (PAMPs). TLRs: toll-like receptors.

The biological significance of CD24-Siglec interaction was first revealed when Chen et al. (23) demonstrated that CD24-Siglec G/10 forms tri-molecular complex with HMGB1 and heat-shock proteins (HSPs), the prototypical danger-associated molecular patterns (DAMPs or danger signal). The concept of danger signal was first proposed to describe inflammatory stimuli released upon tissue injuries or cellular stress (29). Dendritic cells with mutations of either *CD24* or *Siglecg* genes enhanced production of inflammatory cytokines to DAMPs such as HMGB1 and HSP70, but not to pathogen-associated molecular patterns (PAMPs), such as lipopolysaccharides and double-stranded RNA. Targeted mutations of *CD24* or *Siglecg* in the mice fatally exacerbated inflammatory responses to acetaminophen-induced necrosis of hepatocytes without affecting inflammatory responses to PAMPs. Based on these data (23), the author proposed that CD24-Siglec 10 discriminate between PAMPs and DAMPs (23, 30) (**Figure 1**).

Pattern recognition is a pillar of contemporary immunology. As proposed by Janeway, innate immunity based on pattern recognition allowed the immune system to sense infections and launch innate immune response, leading to adaptive immune response (31–34). With the identification of pattern recognition receptors and their ability to recognize both DAMPs and PAMPs (35–39), it was difficult to explain how the pattern recognition allows self-nonself discrimination. The discovery that CD24-Siglec G interaction discriminates DAMPs from PAMPs provide a framework by which the self and non-self discrimination can be achieved by the innate immune system. Thus, while responses to PAMPs was unconstrained by the CD24-Siglec interaction, those to DAMPs was restrained by the interaction, allowing minimal inflammatory response to cell death under physiological conditions (30) (**Figure 1**).

## CD24 in graft vs host diseases

Cellular stress and death are generally physiological and inflammatory response to DAMPs has been shown to beneficial for wound-healing (40, 41). However, massive cell death that occurs as part of cancer therapy can lead to undesirable consequences. Therefore, regulation of host response to cell death can be explored to enhance safety and efficacy of cancer therapy.

Due to graft vs leukemia (GVL) effect, bone marrow transplantation (BMT) is a curative therapy for refractory hematological malignancies (42, 43). Unfortunately, BMT often associates with immune destruction of host tissue by the immune cells in the graft, a pathological condition called graft vs host diseases (GVHD). Given the massive cell death associated with BMT, it was of interest to evaluate whether CD24-Siglec G/10 pathway contributes to pathogenesis of GVHD.

Toubai et al. reported that conditioning for BMT by irradiation reduced expression of Siglec G on the dendritic cells, leading to an increased expression of inflammatory cytokines and co-stimulatory molecules on the dendritic cells (13). Targeted mutations of either *CD24* or *Siglecg* in the hematopoietic cells greatly exacerbated GVHD, while treatment with CD24Fc, a fusion protein consisting of extracellular domain of CD24 and IgG1 Fc, prevented the development of GvHD by targeting the Siglec G on the hematopoietic cells (13). Importantly, CD24Fc suppressed GVHD without negatively impact GVL and that CD24Fc can suppress DAMP-mediated T cell activation by interacting with Siglec G (14).

Based on the compelling preclinical data, OncoImmune, Inc. launched a randomized, prospective, multi-site, placebo-controlled phase 2a clinical trial to investigate safety of three dose levels of CD24Fc (or blinded placebo) plus standard GVHD prophylaxis with tacrolimus and methotrexate in matched unrelated donors. The trial enrolled 24 patients (CD24Fc, n=18; Placebo, n=6) with minimum follow up of one year. In this translational phase 2a trial, administration of CD24Fc was safe and tolerable. The grade 3-4 GVHD-free survival was 94% at 180 days post BMT in patients receiving treatment with CD24Fc versus 50% in pts receiving placebo (HR 0.1; 90% CI 0.0-0.7) (44). A dose expansion Phase IIb study has since been completed with data to be reported soon.

## CD24 in immunotherapy-related adverse events

Immunotherapy has provided curative treatment for multiple cancer indications. In analogous to BMT, the efficacy of immunotherapy has been limited by immunotherapy-related adverse events (irAE). Given the massive death of cancer cells and normal cells in immunotherapy and conventional cancer therapy, it is of interest to investigate whether irAE is regulated by CD24-Siglec interactions. As the first-step to address this issue, Liu et al. tested the effect of CD24Fc on irAE and tumor responses (45). Using animal model of irAE (46), the authors demonstrated that CD24Fc ameliorate irAE caused by clinically used anti-CTLA-4 mAb, including multiple organ inflammation and animal survival. Surprisingly, CD24Fc not only preserve cancer therapeutic effect of anti-CTLA-4 and anti-PD-1 antibodies, but promoted tumor rejection in some cancer models, concurrent with reducing the frequency of regulatory T cells among CD4 cells and preventing exhaustion of CD8 T cells in the tumor microenvironment (45). Additional studies are needed to understand how CD24-Siglec pathway regulates tumor microenvironment.

## CD24 in COVID-19 and acquired immunodeficiency syndrome

Although early studies demonstrate that CD24-Siglec G/10 interaction controls inflammatory response to DAMPs but not PAMPs, host responses to pathogenic infections can be affected by this interaction because most infections cause cell death and thus may trigger inflammatory response to DAMPs. Moreover, many pathogens have been shown to disrupt CD24-Siglec G/10 interaction, either by down-regulation of Siglec G/10 (47) or by desialylation of CD24 (9). Preclinical studies have shown that CD24Fc protected non-human primates against acquired immunodeficiency syndrome (AIDS) caused by the simian immunodeficiency virus, including diarrhea and pneumonia (48, 49).

Based on the strong therapeutic effect in the non-human primate against AIDS as well as the safety and clinical activities of CD24Fc in healthy volunteers and BMT patients, OncoImmune, Inc. launched a randomized, double-blind, placebo-controlled, phase 3 study at 9 medical centers in the US testing safety and clinical efficacy of CD24Fc for hospitalized COVID-19 patients who needs oxygen support. The primary efficacy endpoint is time to clinical improvement from requiring oxygen support to independent of oxygen support during 28 days of study period. The data demonstrated that CD24Fc is well tolerated and significantly accelerates clinical improvement, by more than 60%, of hospitalized patients with COVID-19 who are receiving oxygen support (HR=1.61, 95% CI 1.16-2.23; P=0.0028) (50). Biomarker studies reveal that CD24Fc systematically repress inflammatory response in the COVID-19 patients (51). Taken together, the data demonstrated that targeting inflammation in response to tissue injuries may provide a therapeutic option for patients hospitalized with COVID-19.

Consistent with clinical activities of CD24Fc, HMGB1 is elevated in plasma of COVID-19 patients (52). More importantly, RNAseq analysis of lung tissue from healthy control and severe COVID-19 patients revealed selective reduction of *SIGLEC10* mRNA without affecting expression of other *SIGLECS* (47). More recently, Shapira et al. reported in a non-randomized study that Exo-CD24, the CD24-containing exosomes, appeared to reduce inflammatory markers and cytokine/chemokine while accelerated recovery of hospitalized COVID-19 patients (53).

## CD24-Siglec interaction and metabolic disorders

While the initial studies focused on the CD24-Siglec 10/Siglec G interaction, CD24 interacts with multiple Siglecs (12, 54). Given the promiscuous nature of CD24-Siglec interactions, genetic studies are necessary to understand contribution of different Siglecs in different disease models.

Metabolic disorders are among the most common threat to human health. While it is clear that chronic inflammation is a root cause, the host factors that regulate the chronic inflammation remain largely unidentified. A Phase I clinical study revealed unexpected activity of CD24Fc in reducing low-density lipoprotein levels in the plasma, which is consistent with broad down-regulation of inflammation-related genes (12). To confirm the rule for CD24 in metabolic disorder, Wang et al. compared CD24^+/+^ and CD24^-/-^ littermate mice for their development of metabolic disorders, including obesity, dyslipidemia, insulin resistance, and nonalcoholic steatohepatitis (NASH) in response to high-fat feed diet and aging (12). The data demonstrated a critical role for CD24 in suppressing metabolic disorders. To identify CD24 receptor responsible for the metabolic disorders, the authors produced a panel of mouse strains with single or combined mutations of one or more *Siglec* genes, including those that encode CD22, CD33, Siglec E, Siglec F, Siglec G or Siglec H proteins. Among all genes tested, only *Siglece* mutation phenocopied that of *CD24*, suggesting that Siglec E is the functional receptor for CD24 in protection against metabolic disorders. This hypothesis is confirmed by direct and sialylation-dependent CD24-Siglec E interaction, requirement of CD24 in either the same (cis) or separate cells (trans) in constitutional activation of Siglec E. More importantly, in a Siglec E-dependent manner, CD24Fc effectively suppressed obesity, dyslipidemia, insulin resistance, and NASH in multiple mouse models.

## Concluding remarks

**Figure 2:**
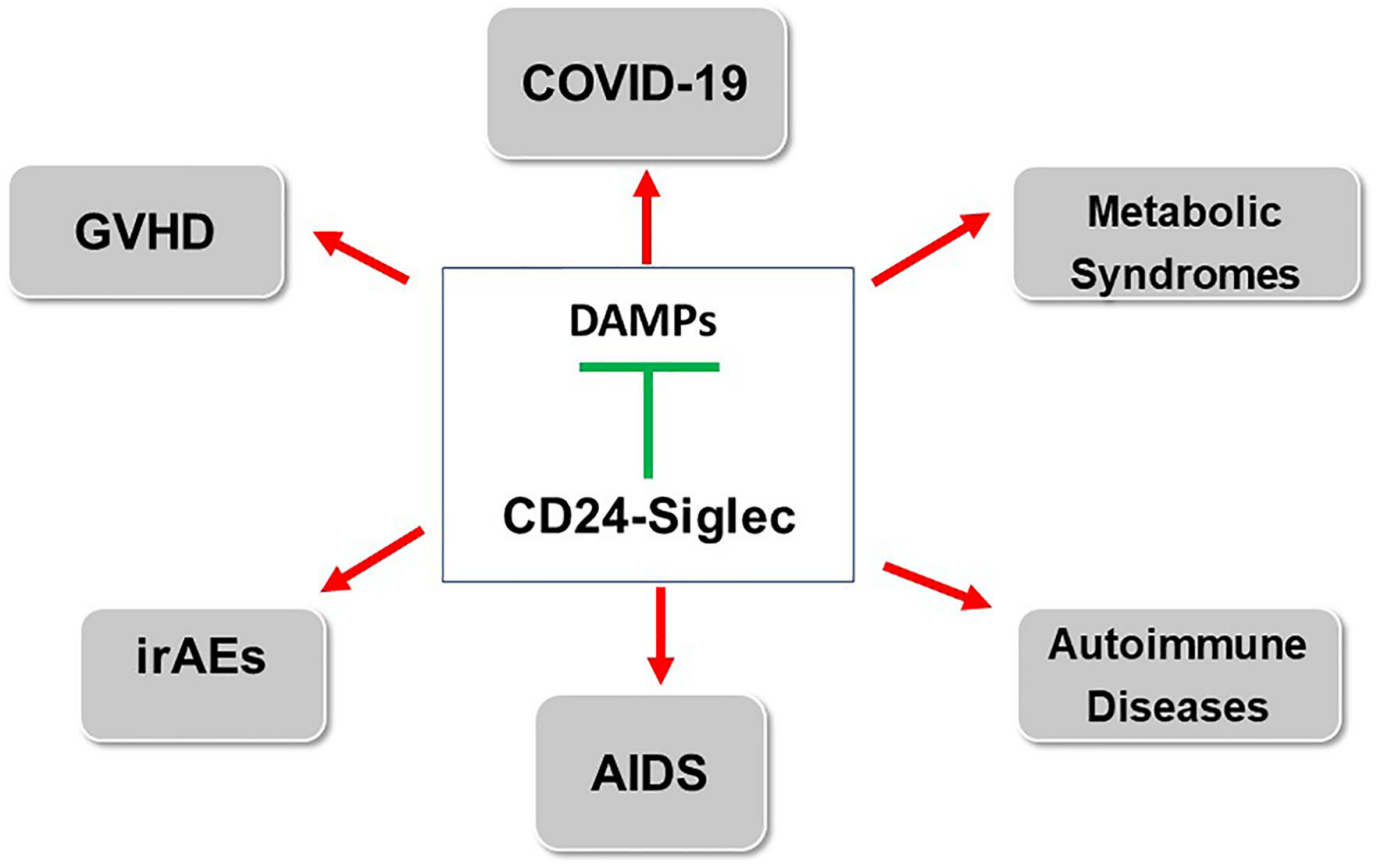
Stimulating CD24-Siglec pathway for treatment of inflammatory and autoimmune diseases. The arrows indicate diseases in which either clinical (GVHD, COVID-19) or preclinical data (IrAE, AIDS, metabolic syndrome and autoimmune diseases) have been reported. GVHD: Graft vs host diseases; AIDS: acquired immunodeficiency syndrome; irAE: immunotherapy-related adverse events.

As an endogenous ligand for Siglecs, CD24 has been implicated as a dominant suppressor of inflammatory responses in a number of pathological conditions. The biological significance of this pathway has been expanded into the realm of both sterile and non-sterile inflammations, and the concept has been confirmed in both preclinical study and clinical trials (**Figure 2**). In addition to inflammation, CD24 has been implicated in oncogenesis and tumor evasion of host immunity. While this review has focused on how this pathway maybe fortified to treat or prevent inflammatory diseases, the role for CD24 in cancer therapy has also attracted significant interest. Thus CD24-Siglec pathway has a vast potential for translation to patient cares.

## Author contributions

YL and PZ cowrote this mini review, which is in response to invitation for “CD24 in the Regulation of Cellular Development and Disease” (edited by Geraldine Cambridge, Sherri Christian). All authors contributed to the article and approved the submitted version.
